# Angular dependent anisotropic terahertz response of vertically aligned multi-walled carbon nanotube arrays with spatial dispersion

**DOI:** 10.1038/srep38515

**Published:** 2016-12-14

**Authors:** Yixuan Zhou, Yiwen E., Xinlong Xu, Weilong Li, Huan Wang, Lipeng Zhu, Jintao Bai, Zhaoyu Ren, Li Wang

**Affiliations:** 1 State Key Lab Incubation Base of Photoelectric Technology and Functional Materials, International Collaborative Center on Photoelectric Technology and Nano Functional Materials, School of Physics, Northwest University, Xi’an 710069, China; 2 Institute of Physics, Chinese Academy of Sciences, Beijing 100190, China

## Abstract

Spatial dispersion effect of aligned carbon nanotubes (CNTs) in the terahertz (THz) region has significance for both theoretical and applied consideration due to the unique intrinsically anisotropic physical properties of CNTs. Herein, we report the angular dependent reflection of *p*-polarized THz wave from vertically aligned multi-walled CNT arrays in both experiment and theory. The spectra indicate that the reflection depends on the film thickness of vertically aligned CNTs, the incident angle, and the frequency. The calculation model is based on the spatial dispersion effect of aligned CNTs and performed with effective impedance method and the Maxwell-Garnett approximation. The results fit well with the experiment when the thickness of CNT film is thin, which reveals a coherent superposition mechanism of the CNT surface reflection and CNTs/Si interface reflection. For thick CNT films, the CNTs/Si interface response determines the reflection at small incident angles, while the CNTs surface effect dominates at large incident angles. This work investigates the spatial dispersion effect of vertically aligned CNT arrays in the THz region, and paves a way for potential anisotropic THz applications based on CNTs with oblique incidence requirements.

Since the discovery of carbon nanotubes (CNTs) in 1991^1^, there has been increasing interests in the optical properties of aligned CNTs such as in-plane aligned, vertically aligned, and selectively isolated tubes, due to the unique intrinsically anisotropic physical properties and the potential applications in electro-optical devices of these materials^2^,^3^. These polarization dependent absorption, transmission, reflection, and resonant scattering properties have been studied by spectroscopic techniques ranging from terahertz (THz) and far-infrared^4^,^5^,^6^,^7^,^8^,^9^,^10^,^11^, to infrared and visible^12^,^13^,^14^,^15^,^16^ spectroscopy as well as the Raman^17^,^18^ and pump-probe spectroscopic techniques^4^,^5^,^6^,^7^. The results of these measurements have convincingly demonstrated that the anisotropic absorption and scattering will reach the maximum and the minimum in the opposite extreme conditions when the light is parallel and perpendicular to the nanotube axis, respectively.

Technically speaking, most of the above mentioned experiments are performed at normal incidence, in which condition the anisotropic response of light can be achieved thank to the manufacturing methods for in-plane aligned structures of CNTs. The vertically aligned CNTs can be regarded as an uniaxial medium, which demonstrates some novel phenomena such as the birefringent and biabsorption effect^16^,^19^, asymmetric properties of waves propagating upward and downward with respect to interfaces^20^. However, due to the measurement technique limitation, the response of vertically aligned CNTs at oblique incidence receives less research than that at normal incident condition. The study of these oblique incidence dependent properties are of considerable interest for not only polarizer but also absorber applications based on CNTs^12^,^13^,^15^.

THz wave region locates at the “gap” between the microwave region and the infrared region with broad prospect of applications^21^. The oblique incidence dependent properties of vertically aligned CNTs are still unexploited in this region. Such studies are significant for both theoretical and applied consideration in THz region. Firstly, the vertically aligned CNTs afford a unique platform for the observation of novel phenomena, which cannot be achieved in nature materials. Such as the spatial dispersion effect^22^, which has been proposed to be found in wire media^23^, is possible to be observed in aligned CNTs in THz region. Secondly, the verification of theory method to reveal the mechanism of CNTs’ reflection phenomena can be extended to the analysis of other anisotropic material such as metamaterials. Thirdly, the clarification to the oblique incidence dependent property will support the development of THz applications such as THz generators^24^,^25^, detectors^26^ and polarizers^27^,^28^,^29^,^30^,^31^,^32^ etc. based on CNTs.

In this paper, reflection of *p*-polarized THz waves from vertical CNT arrays is measured by a THz time-domain spectroscopy (THz-TDS) system with a variable angle experimental con. Vertically aligned CNT arrays with different thicknesses are synthesized on high-resistance silicon (HR-Si) by a floating catalytic chemical vapor deposition (CVD) method. The time-domain spectra show that the existence of CNT layer totally changes the reflection from bare HR-Si substrate, and the Fourier transform spectra in frequency domain further indicate that these changes depend on the thickness of CNTs, the incident angle and the frequency. We firstly used an effective impedance method combined with the Maxwell-Garnett (MG) model based on the spatial dispersion of aligned CNTs to explain the experiment phenomena. Theory calculations fit well with the thin CNT film condition, and can also follow the tendency of thick CNT film condition. The results demonstrate that the reflection from thin CNT films is governed by a coherent superposition effect of the CNT surface reflection and the CNTs/Si interface reflection. For thick CNT films, the film absorption dependent interface effect is the main factor at small incident angles, while the surface effect plays the key role at large incident angles. This work investigates the spatial dispersion effect of vertically aligned CNT arrays in THz region, and paves a way for potential THz applications based on CNTs or similar anisotropic materials at oblique incidence.

## Results and Discussion

### Morphology characterization of the CNT samples with SEM and TEM

**Figure 1:**
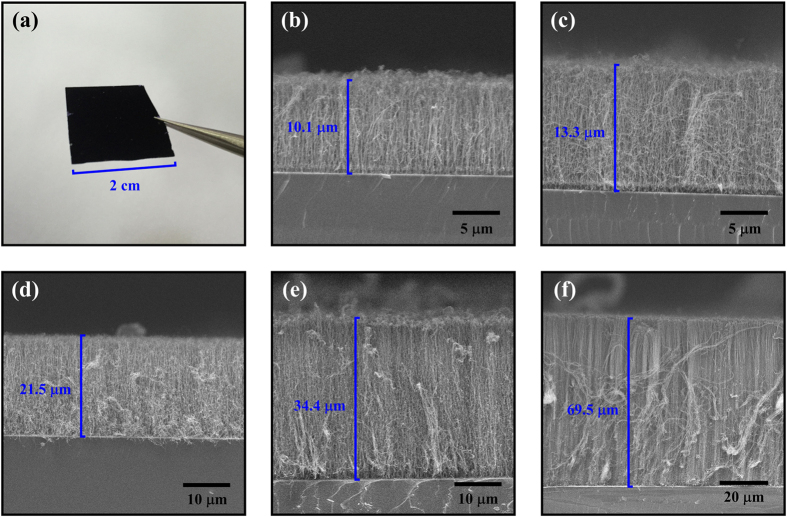
(**a**) Photography of a CNT array on HR-Si sample. SEM cross-section images of the CNT arrays with different growth time: (**b**) 1.5 min, (**c**) 3.0 min, (**d**) 5.0 min, (**e**) 7.0 min, (**f**) 10.0 min.

CNT arrays with different thickness are grown on HR-Si substrates by floating catalytic CVD as described in the Methods part. Figure 1(a) shows the photograph of a typical aligned CNT film (growth time: 5.0 min. CNT thickness: 21.5 μm) with an area of about 2 × 2 cm^2^ grown on a HR-Si substrate. It suggests that our fabrication method is beneficial to maintain the macroscopically uniformity of a large area CNT film, so as to meet the millimeter-scale THz spot size in the reflection measurement. In order to confirm the micro-morphology of the CNT arrays, fractured edges of the samples have been characterized by a scanning electron microscopy (SEM, FEI Quanta 400 ESEM-FEG) as shown in Fig. 1(b–f). It can be seen that all these CNT films are composed of aligned CNT arrays vertical to the substrate surfaces. However, the blue lines in the films’ cross sections show different thicknesses. That is because these CNT array samples are made by increased growth time. For the samples from Fig. 1(b) to (f), the growth time is 1.5, 3.0, 5.0, 7.0 and 10.0 min, respectively. Correspondingly, the length of CNTs become longer and longer along with the elevated growth time. Finally, the thicknesses of the films shown in the above mentioned figures are measured to be 10.1, 13.3, 21.5, 34.4, 69.5 μm, respectively. For convenience, we call these samples CNT1–5 for short.

**Figure 2:**
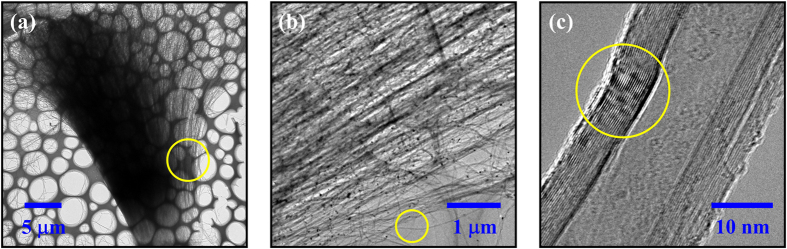
TEM images of CNTs peeled off from the 7 min grown sample, with different magnifications. (**a**) A cluster of CNTs; (**b**) Aligned CNTs; (**c**) Cross-section of a single CNT.

Furthermore, the composition units CNTs, which cannot be well distinguished in the SEM images, have been analyzed with routine transmission electron microscope (TEM, Tecnai G2 F20 S-TWIN, point resolution: 0.24 nm, line resolution: 0.14 nm) characterization. Figure 2(a) shows a whole microstructure of a CNT cluster peeled off from the 7 min grown sample. The lengths of these CNTs are about 30 μm, which match well with the SEM result in Fig. 1(e). Figure 2(b) shows a zoom–in part from the yellow circle area in Fig. 2(a). It can be seen that some small particles are distributed in the CNT array. They are catalyst Fe particles which are induced by the CVD process, as discussed in our previous work^33^. From the scope of yellow circle in Fig. 2(b), a single CNT is randomly selected and shown in Fig. 2(c). It clearly demonstrates that the sample is multi-walled, with diameter of about 28 nm and tube wall thickness of around 10 nm. What is more, the yellow line circled area shows that the tube wall is formed by ~24 carbon layers. In addition, the film thickness dependent Raman data have also been measured, the results show that these CNTs have similar good quality. Details can be found in the first part of the Supplementary Information.

### THz time-domain spectroscopy results

**Figure 3:**
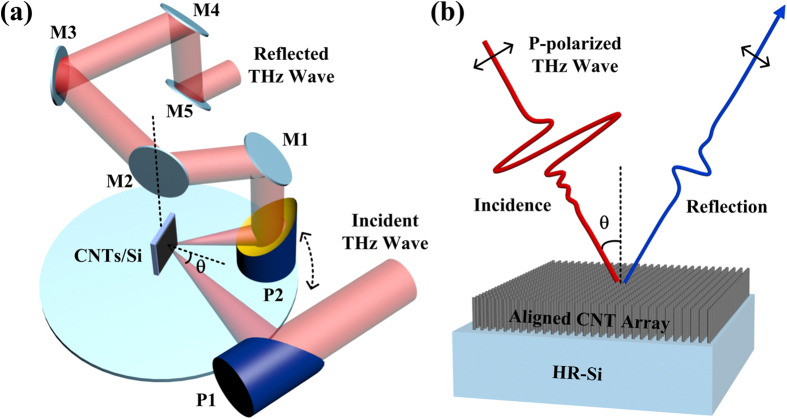
Schematic illustration of (**a**) the reflection geometry setup for the angle-dependent THz-TDS measurement; (**b**) the experiment arrangement of aligned CNT array sample with a *p*-polarized THz wave incident.

A custom-designed angle-dependent THz-TDS is used to perform the reflection measurement. THz experimental geometry is shown in Fig. 3(a) and specifically described in the Methods part. As shown in Fig. 3(b), an aligned CNT array perpendicular to the HR-Si substrate surface is placed against the THz wave incidence in the measurement. The THz wave is *p*-polarized, and the incident angle *θ* is set to 15°, 30°, 45°, 60° and 70°, respectively.

**Figure 4:**
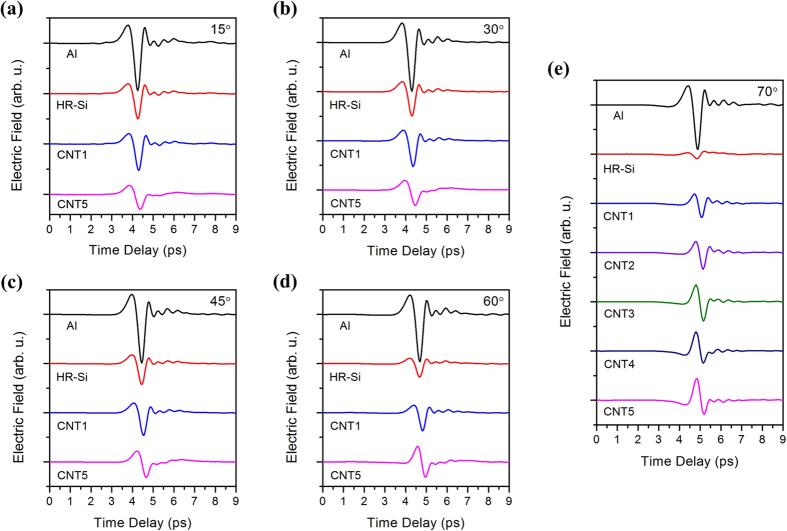
Reflected THz pulses from Al, HR-Si, CNT arrays on HR-Si substrates, with variable incident angles of (**a**) 15°, (**b**) 30°, (**c**) 45°, (**d**) 60° and (**e**) 70°.

Figure 4 shows the original THz time domain signals reflected from the surface of aluminum (Al) mirror and HR-Si as references, and CNT array samples (CNT1–5) on HR-Si substrates. In Fig. 4(a–f), spectra with elevated incident angles 15°, 30°, 45°, 60° and 70° are shown, respectively. In all case, the reflection pulses from Al mirror have similar shapes and amplitudes. That is because Al mirror can reflect all the incident THz wave without energy loss. Then, from the reflection pulses of HR-Si, it can be seen that the amplitude declines obviously with the increased incident angle. The thickness of HR-Si is 500 μm, the substrate internal reflection (the first reflection from bottom face of HR-Si will appear after ~10 ps) is excluded in the time domain window shown here. So the pulses in Fig. 4 are reflected from the front surface of HR-Si. Furthermore, the reflection decline of HR-Si can be attributed to the Brewster angle *θ* = arctan 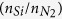 ≈ 73.7° (*n*_*Si*_ = 3.418^34^, 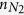
=1).

The most interesting phenomena are the changes of reflection from CNTs. The thickest sample is 69.5 μm (CNT5). Thus the reflection from top surface of CNT array and the reflection from bottom CNT/HR-Si interface cannot be distinguished within ~1 ps. Compared with the reference pulse from HR-Si, no obvious decline of the pulse amplitude from CNT1 has been observed with the rise of incidence angle. Oppositely, the amplitude has been enhanced with thicker CNT array (CNT5). Meanwhile, the shapes of pulses from CNTs at large incident angles become different with those at small incident angles. At a same incident angle below 45°, the shape of pulses from CNTs are similar to that from HR-Si, and the amplitudes of pulses from CNT1 sample are a little larger than those from HR-Si and CNT5. At a large incident angle over 60°, although the pulse amplitudes of HR-Si decline obviously, the pulses from CNT1 change very small, and the pulses from CNT5 even become large. Besides, the phase dependent pulses shape changes gradually with the increase of the film thickness as shown in Fig. 4(e).

### THz spectroscopy in the frequency domain after Fourier transformation

**Figure 5:**
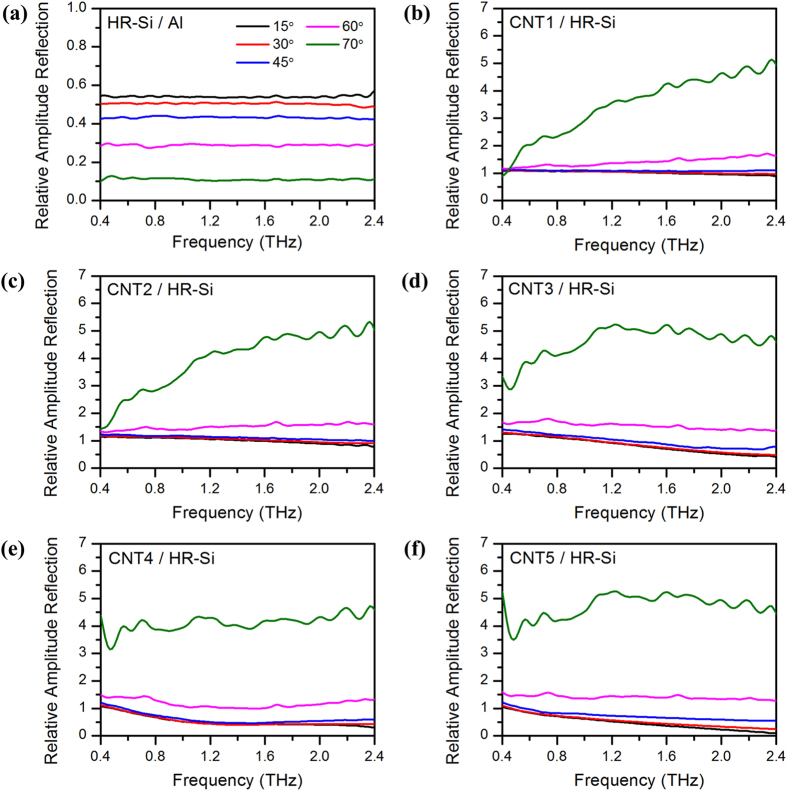
(**a**) Frequency dependent relative amplitude reflection of HR-Si compared with Al. (**b–f**) Frequency dependent relative amplitude reflection of CNT1, CNT2, CNT3, CNT4 and CNT5 compared with HR-Si, respectively. Reflection with elevated incident angles 15°, 30°, 45°, 60° and 70°, is shown as lines with different colors.

In order to reveal the underline mechanisms, Fourier transformation of the THz pulses from 1 to 9 ps in time domain have been performed, and the resulting frequency-dependent THz relative amplitude reflection spectra are shown in Fig. 5 with elevated incident angles 15°, 30°, 45°, 60° and 70°. The relative reflection in Fig. 5(a) is obtained by amplitude reflected from HR-Si compared with that from Al, while the reflection in Fig. 5(b–f) are by amplitudes reflected from CNT samples (CNT1–5, respectively) compared with those from bare HR-Si.

From Fig. 5(a), all the spectral lines in different incident angles are flat and featureless over a broad bandwidth from 0.4 to 2.4 THz. It proves that the refractive index of HR-Si in this region is a frequency independent constant. When the refractive index value 3.418 is used^34^, the theoretically calculated relative amplitude reflection of HR-Si is 0.54 (15°), 0.50 (30°), 0.42 (45°), 0.28 (60°) and 0.10 (70°), respectively. These values fit well with the experimental results in Fig. 5(a).

From Fig. 5(b–f), changes of the relative amplitude reflection of CNT arrays can be observed more clearly. Generally speaking, the relative amplitude reflection of CNTs rises with the elevation of incident angle, and the increase is inconspicuous at small incident angle while dramatic at large angle. What is more, the response are also CNT film thickness dependent and frequency dependent. When the incident angles are below 45°, the relative amplitude reflection of CNT1 is always approximate 1 as shown in Fig. 5(b). In high frequency region, this value drops slowly. This decline phenomenon can be amplified with thick CNT samples. From Fig. 5(c–f), although the relative reflection of a thicker CNT array is still close to 1 in low frequency region, the values become weaker and weaker in high frequency region. Take the thickest CNT5 as an example, the relative amplitude reflection becomes no more than 0.1 at 2.4 THz as shown in Fig. 5(f).

When the incident angle becomes larger than 60°, sample thickness and frequency dependent reflection enhancement will appear. From Fig. 5(b), in small incident angle conditions, the reflection rises with the elevated frequency. Especially for the 70° incidence, an obvious increasing reflection with the increasing of frequency can be observed. Moreover, there is another phenomenon that the reflection also changes with the CNT film thickness. As shown in Fig. 5(c–f), along with the increase of the CNT forest height, the frequency dependent of reflection becomes less distinct. When the thickness is large enough such as CNT4 and CNT5, the reflection spectra seem to be flat in frequency domain. These oblique incidence dependent reflection phenomena is originated from the anisotropic THz dielectric functions of CNT arrays. The underline mechanisms have been revealed in the following part with the help of theoretical calculations.

### Theoretical calculation results

**Figure 6:**
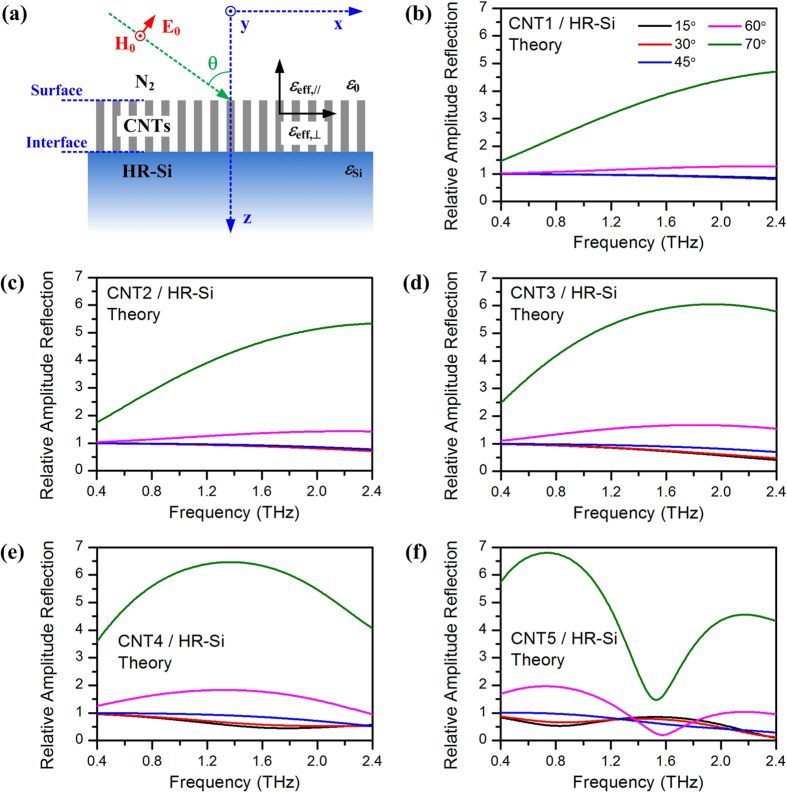
(**a**) Schematic diagram of *p*-polarized THz wave reflected from a CNT array, which is equivalent to an anisotropic layer with effective dielectric constants of *ε*_*eff*,⊥_ and *ε*_*eff*,∥_ for incident THz electric fields perpendicular and parallel to the CNT axial (z-axial). (**b–f**) Theoretically calculated relative amplitude reflection of CNT1, CNT2, CNT3, CNT4 and CNT5 compared with HR-Si, respectively. Reflection with elevated incident angles 15°, 30°, 45°, 60° and 70°, is shown as lines with different colors.

A schematic view of a vertically aligned CNT array on HR-Si is shown in Fig. 6(a), where a Cartesian coordinate system (x, y, z) is defined. The layer is composed of aligned vertical CNTs in the N_2_ atmosphere. In order to calculate the reflection from this layer theoretically, we consider it as an uniaxially anisotropic film (thickness *d*) between N_2_ atmosphere (dielectric constant *ε*_0_ = 1) and HR-Si substrate (dielectric constant *ε*_*Si*_ = 3.418^2^). So the permittivity of the layer can be described as *ε*_*x*_ = *ε*_*y*_ = *ε*_*eff*,⊥_ and *ε*_*Z*_ = *ε*_*eff *_∥__. The former *ε*_*eff*,⊥_ represents the effective dielectric constant for incident THz electric field perpendicular to the axial direction of CNT (z-axial), while the later *ε*_*eff *∥_ represents the effective dielectric constant for incident electric field parallel to the axial. With the consideration of the spatial dispersion effect, for an oblique incident *p*-polarized THz wave with angle *θ* by solving Maxwell’s equations, the normal component of the wave vector *k*_Z_ and the transverse wave impedance Z_*CNTs*_ in CNT layer can be obtained as^20^,^35^:


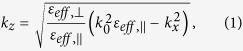



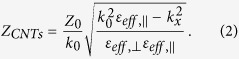


where *k*_0_ is the wave vector in free space, Z_0_ = 377 Ω is the impedance of free space. Being related with the incident angle, the transverse component of wave vector in the CNT layer is:


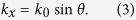


Because the experimental results have eliminated the internal reflections from the HR-Si substrate (as described in the time-domain data), the effective impedance of CNTs/HR-Si system can be written as^36^:


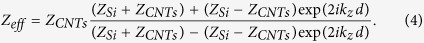


Based on the above equations, the reflection coefficient from CNTs/HR-Si (*r*_*CNTs*_) and HR-Si (*r*_*Si*_) can be given as 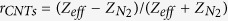 and 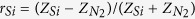, where 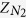 and *Z*_*Si*_ are the transverse wave impedance in N_2_ atmosphere (free space) and HR-Si substrate, respectively. If we consider the reflection from CNT layer surface and CNTs/HR-Si interface separately, the first reflection from CNT layer *r*_*surface*_ and the second reflection from CNTs/HR-Si interface *r*_*interface*_ can be given as:


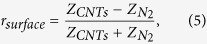



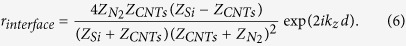


Based on these reflection coefficients, the relative amplitude reflection of CNTs/HR-Si, the relative amplitude reflection from CNT layer surface and CNTs/HR-Si interface can be given as |*r*_*CNTs*_|/|*r*_*Si*_|, |*r*_*surface*_|/|*r*_*Si*_|, and |*r*_interface_|/|*r*_*Si*_|, respectively. And the phase difference of reflection from CNT layer surface and CNTs/HR-Si interface can be obtained from calculating the phase angle of *r*_*surface*_/*r*_interface_. More details about the theory method for our reflection calculation can be found in the second part of the Supplementary Information.

On the other hand, the effective dielectric constant *ε*_*eff*,⊥_ and *ε*_*eff*,||_ should be provided. Here we simplify CNTs as cylinders with the same radius and length in vacuum and calculate them with an effective medium approach MG approximation (Eqs S11 and S12 in the Supplementary Information)^14^,^37^. The dielectric constant of CNTs themselves are evaluated with a combination of a Drude term and localized Lorentzian absorptions (Eq. S13 in the Supplementary Information)^8^,^9^. After contrasting and optimizing the parameters of these equations, we obtain the theoretically calculated relative amplitude reflection of CNT1 to CNT5 and show them in Fig. 6(b–f), respectively. More calculation details and parameters about the effective medium model can be found in the third part of the Supplementary Information.

By contrasting the experiment results in Fig. 5(b–f) and the theory results in Fig. 6(b–f) correspondingly, it can be seen that the theoretical lines fit well with the experiment in thin CNT layer conditions. Especially for CNT1 to CNT3, the similar reflection at small incident angles below 45°, the dramatic enhancement at large incident angles, and even the thickness dependent tendency, are well reflected in the theoretical results as shown in Fig. 6(b–d). However, although theoretical lines still follow the tendency of experiment, obvious differences can be observed for thick samples such as CNT4 and CNT5. As shown in Fig. 5(e) and (f), the reflection spectra are all flat with no features. However, the lines in Fig. 6(e) become curving, and peaks and valleys appear in Fig. 6(f).

As we discussed before, the internal reflection from HR-Si substrate has been eliminated in time domain window, but the reflection from the top surface of CNT array and CNTs/HR-Si interface are involved (as shown in Fig. 4). Thus the coherent superposition of THz wave in CNT films is involved in the reflection spectra. Under the assumption of homogeneous anisotropic film, phase relationship induced interference must be considered because the film thickness of CNT array is several dozens of micrometers, which is comparable to the THz wavelength. This is the reason for the appearance of the interference peaks and valleys in the calculated results as shown in Fig. 6(e) and (f). However, these phenomena have not been obtained in the experimental results as shown in Fig. 5(e) and (f). In order to reveal the underline mechanisms, we make a further calculation and discussion in the following part.

### Discussion on the fitting condition of the theory and experiment results

**Figure 7:**
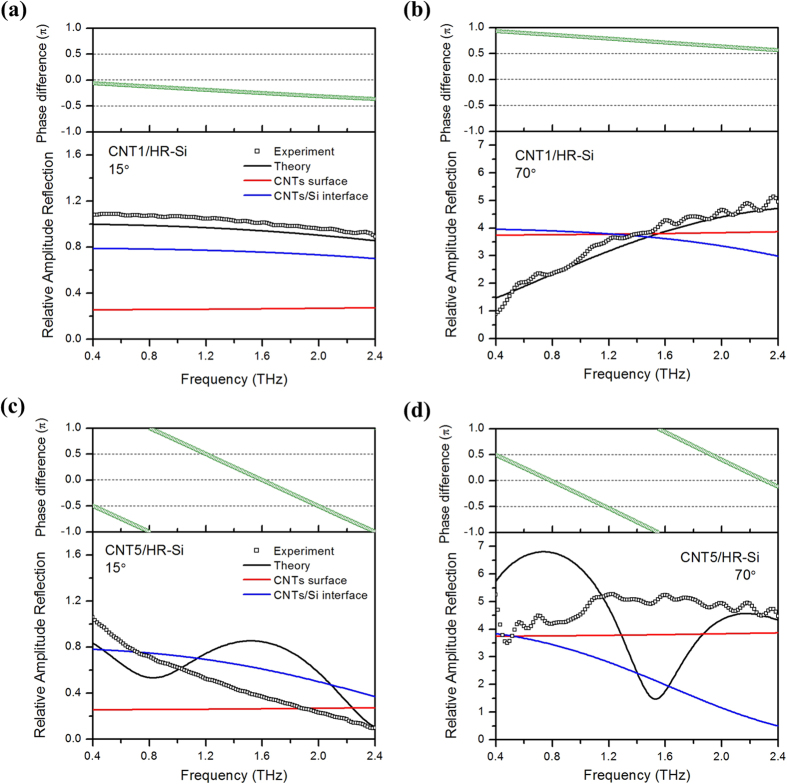
Frequency dependent relative amplitude reflection and phase difference of (**a**) CNT1 at 15°, (**b**) CNT1 at 70°, (**c**) CNT5 at 15° and (**d**) CNT5 at 70°. In each top figure, green lines is the theoretical phase difference of reflection from CNT layer surface and CNTs/HR-Si interface. In each bottom figure, Black circle points represent the experiment values, black lines are the corresponding theoretical relative amplitude reflection, red and blue lines are the theoretically calculated relative amplitude reflection from CNT layer surface and CNTs/HR-Si interface, respectively.

As typical thin and thick films, CNT1 and CNT5 are selected and their frequency dependent relative amplitude reflection and phase difference spectra are shown in Fig. 7. Meanwhile, 15° and 70° are used to represent the small and large incidence angle conditions. In the bottom figures, theoretically calculated results are shown as black lines, while the experimental values are black circle points. Additionally, we have also calculated the relative amplitude reflection from CNT layer surface and CNTs/HR-Si interface separately. The former is shown as red lines and the latter is shown in blue. In the top figures, the phase difference of reflection from CNT layer surface and CNTs/HR-Si interface is shown in green. Detailed calculation method can be found in the Supplementary Information.

From Fig. 7(a) and (b), it can be seen that for both 15° and 70° incidence conditions of CNT1, the theory lines agree very well with the experiment. It must be clarified that the black lines are approximate to the coherent superposition of the red and blue lines, and their phase differences are shown in the top figures in green. When the incidence angle is 15°, the phase difference is small, so the final result is close to a direct superposition of the CNT surface and CNTs/HR-Si interface reflection. More importantly, the results suggest the interface reflection account for a large share in this case. It also agrees well with the approximate relationship between CNT1 and HR-Si as shown in Fig. 4(a). When the incident angle becomes 70°, both the relative amplitude reflection from the CNT surface and the CNTs/HR-Si interface have flat frequency dependency and are largely enhanced in value as shown in Fig. 7(b). However, the total reflection shows an obvious frequency dependent increase. It can be attributed to the coherent superposition effect decided by the phase difference, which changes from almost π at 0.4 THz to around π/2 at 2.4 THz. The region before the point ~1.5 THz (2π/3) is constructive interference, and after this point is destructive interference.

With long CNT5 array, as shown in Fig. 7(c) and (d), it can be seen that although theoretical lines still follow the tendency of experimental results, but obvious peaks and valleys in the theory cannot match the featureless experimental spectra. From the phase difference of CNT surface and CNTs/HR-Si interface reflection, the peaks appear at zero phase points and valleys at −π or π points. This suggest that coherent superposition effect counts for the peak and valley in thick films, which has not been observed in the experiment. This is because the model is within the MG scope where CNT layer is assumed to be homogeneous anisotropic and the various inhomogeneity of forest-like structural features of CNT array have been neglected. Although complete fitting has not been achieved, the changing tendency and mechanism for thick samples can also be revealed based on our theory.

By comparing the reflection of CNT1 (Fig. 7(a) and (b)) and CNT5 (Fig. 7(c) and (d)), it is clear that the reflection from CNT surface has no relation with the film thickness, while the reflection from CNTs/HR-Si interface has a strong dependency. Along with the increase of the film thickness, both the interface reflection at 15° and that at 70° drop obviously with the increasing of frequency. It can be attributed to the absorption effect of CNTs. As a result, the different performances of the total reflection at 15° and 70° can be attributed to the incident angle dependent change of the surface and interface reflection. When the incident angle is 15°, the absorption effect dependent CNTs/HR-Si interface effect plays a leading role, while at the incident angle of 70°, the surface effect dominate.

At last, we give an explanation to the difference between the experiment and theory for thick CNT samples. Inhomogeneity structure induced multi-reflection between separated CNTs has been considered to be an important fact deciding the dark absorber effect of vertically aligned CNTs in the visible-infrared region^38^. Because the wavelength in the THz region is much larger, our model based on homogeneity assumption can fit the thin CNT film condition well. However, with the increase of thickness, structural inhomogeneity induced scattering effects etc. become more significant. So the reflection cannot be seem as a perfect coherent superposition any more.

In conclusion, this work investigates the reflection of vertically aligned multi-walled CNT arrays decided by the thickness of CNTs layer, the incident angle and the frequency. The theoretical calculation fits well with the experiment. It is the first time to investigate the spatial dispersion effect of vertically aligned CNT arrays in THz region. The method might be extend to other anisotropic materials. From the application viewpoint, it paves a way for potential THz applications based on CNTs or similar anisotropic materials with oblique incidence requirements.

## Methods

### Preparation of CNT array samples

CNT arrays are grown on HR-Si substrates (about 2 × 2 cm^2^, 500 μm thick) by floating catalytic CVD^33^. Ferrocene (Fe(C_5_H_5_)_2_) is used as catalyst precursor and acetylene (C_2_H_2_) as carbon source. Firstly, HR-Si substrate is placed in a quartz tube and heated to the temperature 725 °C by a tube furnace under argon (Ar) atmosphere. Secondly, Fe(C_5_H_5_)_2_ is dissolved in xylene (C_8_H_10_) with a concentration of 0.05 g·mL^−1^ and sprayed into the quartz tube by a syringe in a speed of 0.2 mL/min with the help of carrier gases Ar (800 sccm) and H_2_ (200 sccm). At the same time, C_2_H_2_ is also induced into the tube at 30 sccm to increase the carbon source. In order to obtain CNT arrays of different lengths, the growth time is set to 1.5, 3.0, 5.0, 7.0 and 10.0 min, respectively. Lastly, the sample is cooled to room temperature with the protection of Ar atmosphere and collected carefully.

### THz experiments

A custom-designed angle-dependent THz-TDS^39^ is used to perform the reflection measurement. THz experimental geometry is shown in Fig. 3(a). Incident THz wave is focused on the sample with the help of the parabolic mirror P1, while the reflected THz wave is collected by the parabolic mirror P2. In order to obtain different reflection angles, reflection THz wave is first delivered up to a high plane (the plane where M1-M4 are installed), and then delivered back down to the initial plane (the plane where P1, P2, sample and M5 are installed) by reflective mirrors M1-M5. Notice that sample and mirror M2 are coaxial, while mirrors P2 and M1 are fixed on a rotation stage to guarantee the reflection geometry. Besides, the THz wave is generated by a GaAs photoconductive antenna with 70-fs Ti:sapphire laser (Maitai Spectra-Physics, center wavelength 800 nm, average power 30 mW, repetition rate 80 MHz) excitation and detected by electro-optic sampling with a ZnTe (110) crystal. In addition, the humidity is controlled to below 1.5% with the help of dry N_2_.

## Additional Information

**How to cite this article**: Zhou, Y. *et al*. Angular dependent anisotropic terahertz response of vertically aligned multi-walled carbon nanotube arrays with spatial dispersion. *Sci. Rep.* **6**, 38515; doi: 10.1038/srep38515 (2016).

**Publisher's note:** Springer Nature remains neutral with regard to jurisdictional claims in published maps and institutional affiliations.

## Supplementary Material

Supplementary Information
