# Supplementary material for: Angular dependent anisotropic terahertz response of vertically aligned multi-walled carbon nanotube arrays with spatial dispersion
Source: Sci Rep. 2016 Dec 14;6:38515. doi: 10.1038/srep38515 (PMC5155244; doi:10.1038/srep38515)
Supplement: Supplementary Information [file srep38515-s1.doc]

Supplementary information for:

**Angular dependent anisotropic terahertz response of** **vertically aligned multi-walled carbon nanotube arrays with spatial dispersion**

Yixuan Zhou^1^, Yiwen E^2^, Xinlong Xu^1,*^, Weilong Li^1^, Huan Wang^1^, Lipeng Zhu^1^, Jintao Bai^1^, Zhaoyu Ren^1,*^, and Li Wang^2^

^1^State Key Lab Incubation Base of Photoelectric Technology and Functional Materials, International Collaborative Center on Photoelectric Technology and Nano Functional Materials, School of Physics, Northwest University, Xi'an 710069, China.

^2^Institute of Physics, Chinese Academy of Sciences, Beijing 100190, China.

*xlxuphy@nwu.edu.cn (X. Xu) and rzy@nwu.edu.cn (R. Ren)

**1. Raman** **spectrum of vertically aligned CNT arrays**

**
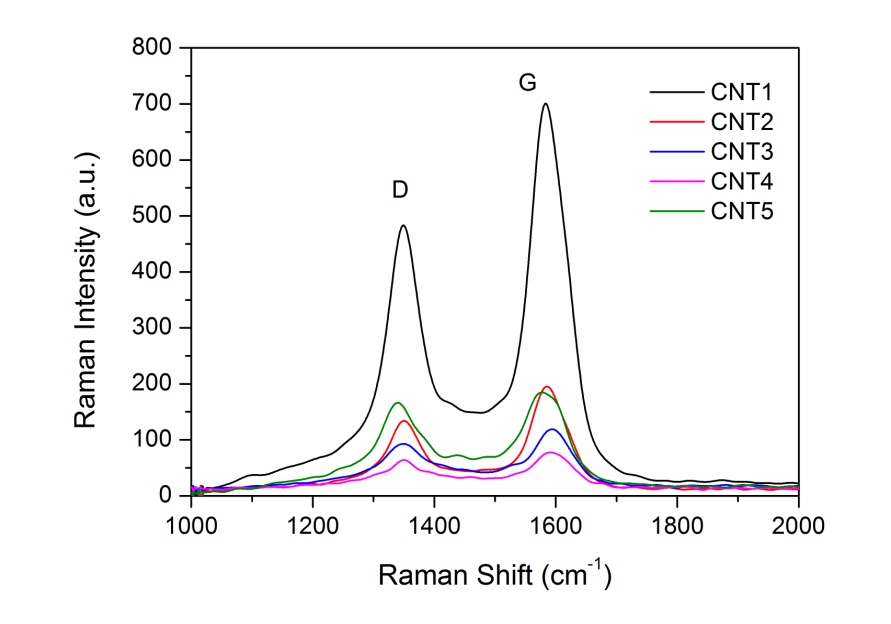
**

**Figure S1.** Raman spectra of CNTs with different thickness.

Raman spectroscopic measurements were carried out with laser excitation wavelength at 514 nm (Laboratory Ram HR800). Thickness dependent Raman spectra of CNT1-5 have been shown in Figure S1. The position of the two bands (D and G) and the value of intensity ratio I_D_/I_G_ are shown in Table S1. These CNT samples have the same growth condition but different growth time. The Raman results show that they have similar good quality. The rise of I_D_/I_G_ suggests that the defects increase and the graphitization degree reduces with the increasing of the growth time. In general, these changes can be ignored in the calculation model for simplification.

| Sample | D band position/cm^-1^ | G band position/cm^-1^ | I_D_/I_G_ |
| --- | --- | --- | --- |
| CNT1 | 1349.6 | 1583.1 | 0.689212 |
| CNT2 | 1349.6 | 1586.1 | 0.685963 |
| CNT3 | 1348.1 | 1595.0 | 0.779647 |
| CNT4 | 1349.6 | 1590.6 | 0.825581 |
| CNT5 | 1340.4 | 1577.1 | 0.899296 |

**Table S1.** Central peak position and intensity ratio of D and G bands.

**2. Theory method for reflection calculation of vertically aligned CNT array**


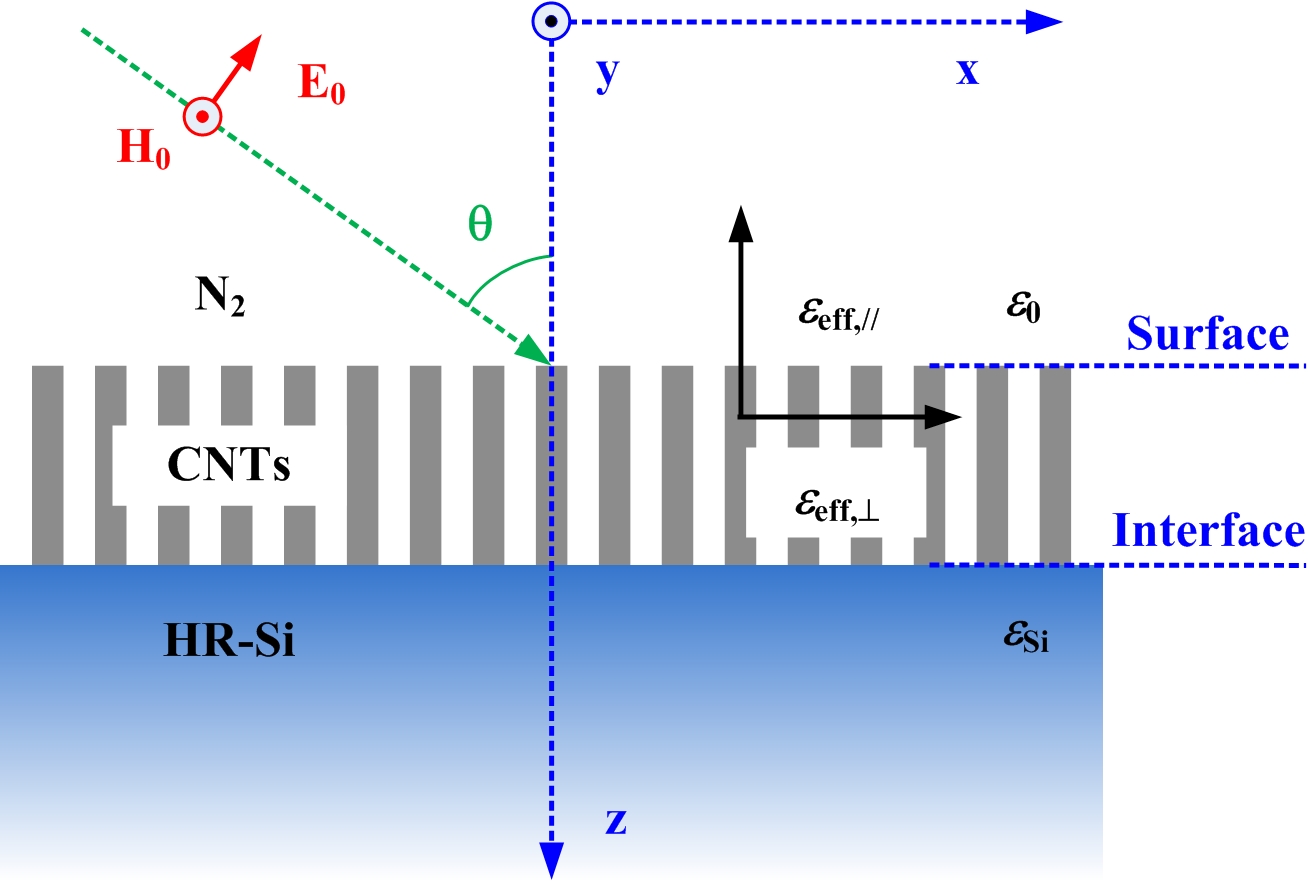


**Figure S2.** Schematic diagram of *p*-polarized THz wave reflected from a CNT array, which is equivalent to an anisotropic layer with effective dielectric constants of $\varepsilon_{eff,\perp}$ and $\varepsilon_{eff,\parallel}$ for incident THz electric fields perpendicular and parallel to the CNT axial (z-axial).

In the coordinate system (x, y, z) as shown in Figure S2 in the main text, the CNT layer is considered as a thin, homogeneous, uniaxially anisotropic film (thickness *d*) between N_2_ atmosphere (dielectric constant $\varepsilon_{0}=1$) and HR-Si substrate (dielectric constant $\varepsilon_{Si}=3.418^{2}$). And the permittivity of the layer can be described as $\varepsilon_{x}=\varepsilon_{y}=\varepsilon_{eff,\perp}$ and $\varepsilon_{z}=\varepsilon_{eff,\parallel}$. The former $\varepsilon_{eff,\perp}$ represents the effective dielectric constant for incident THz electric field perpendicular to the axial direction of CNT (z-axial), while the later $\varepsilon_{eff,\parallel}$ represents the effective dielectric constant for incident electric field parallel to the axial.

With the consideration of the spatial dispersion effect, for an oblique incident (with angle *θ*) *p*-polarized THz wave with space-time dependence of fields as $\exp[-i(\omega t-k_{x}x-k_{z}z)]$, the expression of the electric field can be obtained from solving Maxwell’s equations and eliminating the magnetic field as^1^:

$\left[\begin{matrix} k_{z}^{2}-k_{0}\varepsilon_{eff,\perp} & k_{x}k_{z} \\ -k_{x}k_{z} & -k_{x}^{2}+k_{0}^{2}\varepsilon_{eff,\parallel} \end{matrix}\right]\left[\begin{matrix} E_{x} \\ E_{z} \end{matrix}\right]=0$. (S1)

Where *k*_0_ is the wave vector in free space, and $k_{x}=k_{0}\sin\theta$ is the transverse component of wave vector in the CNT layer. And the normal component of the wave vector in CNT layer can be further evaluated as^1,2^:

$k_{z}=\sqrt{\frac{\varepsilon_{eff,\perp}}{\varepsilon_{eff,\parallel}}(k_{0}^{2}\varepsilon_{eff,\parallel}-k_{x}^{2})}$. (S2)

Furthermore, the transverse wave impedance in CNT layer can be derived as^1,2^:

$Z_{CNTs}=\frac{E_{x}}{H_{y}}=\frac{Z_{0}}{k_{0}}\sqrt{\frac{k_{0}^{2}\varepsilon_{eff,\parallel}-k_{x}^{2}}{\varepsilon_{eff,\perp}\varepsilon_{eff,\parallel}}}$. (S3)

Where $Z_{0}$= 377 Ω is the impedance of free space. Meanwhile, the transverse wave impedance in N_2_ atmosphere (free space) and HR-Si substrate can be given as:

$Z_{N_{2}}=\frac{Z_{0}}{k_{0}}\sqrt{k_{0}^{2}-k_{x}^{2}}$, (S4)

$Z_{Si}=\frac{Z_{0}}{k_{0}}\sqrt{\frac{k_{0}^{2}\varepsilon_{Si}-k_{x}^{2}}{\varepsilon_{Si}^{2}}}$. (S5)

Because the experiment result has eliminated the internal reflections from the HR-Si substrate (as described in the time-domain data in the main text), the effective impedance of CNTs/HR-Si system can be written as^3^:

$Z_{eff}=Z_{CNTs}\frac{(Z_{Si}+Z_{CNTs})+(Z_{Si}-Z_{CNTs})\exp(2ik_{z}d)}{(Z_{Si}+Z_{CNTs})-(Z_{Si}-Z_{CNTs})\exp(2ik_{z}d)}$. (S6)

Based on the above equations, the reflection coefficient from CNTs/HR-Si can be calculated:

$r_{CNTs}=\frac{Z_{eff}-Z_{N_{2}}}{Z_{eff}+Z_{N_{2}}}$. (S7)

And the reference reflection coefficient from HR-Si is:

$r_{Si}=\frac{Z_{Si}-Z_{N_{2}}}{Z_{Si}+Z_{N_{2}}}$. (S8)

If we consider the reflection from CNT layer surface and CNTs/HR-Si interface separately, the first reflection from CNT layer can be given as:

$r_{surface}=\frac{Z_{CNTs}-Z_{N_{2}}}{Z_{CNTs}+Z_{N_{2}}}$. (S9)

And the second reflection from CNTs/HR-Si interface can be given as:

$r_{interface}=\frac{4Z_{N_{2}}Z_{CNTs}(Z_{Si}-Z_{CNTs})}{(Z_{Si}+Z_{CNTs})(Z_{CNTs}+Z_{N_{2}})^{2}}\exp(2ik_{z}d)$. (S10)

Based on these reflection coefficients, the relative amplitude reflection of CNTs/HR-Si can be calculated with $\left|r_{CNTs}\right|/\left|r_{Si}\right|$. The relative amplitude reflection from CNT layer surface and CNTs/HR-Si interface can use $\left|r_{surface}\right|/\left|r_{Si}\right|$ and $\left|r_{interface}\right|/\left|r_{Si}\right|$, respectively. And the phase difference of reflection from CNT layer surface and CNTs/HR-Si interface can be obtained from calculating the phase angle of $r_{surface}/r_{interface}$.

**3. Effective medium model for vertically aligned CNT array layer**

For analyzing the reflection of THz waves from this kind of heterogeneous media, effective medium approach MG approximation has been used to calculate the effective dielectric constants of the film. With CNTs regarding as cylinders with the same radius and length in vacuum, the effective dielectric constants for incident *p*-polarized THz electric fields perpendicular and parallel to the axial direction of CNT (z-axial) can be written as^4,5^:

$\varepsilon_{eff,\perp}(\omega)=\frac{\varepsilon_{\perp}(\omega)+\alpha(\omega)+f[\varepsilon_{\perp}(\omega)-\alpha(\omega)]}{\varepsilon_{\perp}(\omega)+\alpha(\omega)-f[\varepsilon_{\perp}(\omega)-\alpha(\omega)]}$, (S11)

. (S12)

Where $\alpha(\omega)=\varepsilon_{\perp}(\omega)/\varepsilon_{\parallel}(\omega)$, and $f$ is the volume fraction occupied by the cylinders. The other two functions $\varepsilon_{\perp}(\omega)$ and $\varepsilon_{\parallel}(\omega)$ are the dielectric constant components of CNTs themselves for the field perpendicular and along the axis of *z*, respectively. In the THz region, both of them can be given as a combination of a Drude term and localized Lorentzian absorptions by^6,7^:

$\varepsilon(\omega)=\varepsilon_{c}-\frac{\omega_{p}^{2}}{\omega^{2}+i\omega\Gamma}+\sum_{j} \frac{\Omega_{pj}^{2}}{(\omega_{j}^{2}-\omega^{2})-i\omega\Gamma_{j}}$. (S13)

Where $\omega$ is the angular frequency, $\varepsilon_{c}$ is the frequency-independent dielectric constant, $\omega_{p}$ and $\Gamma$ are the plasma frequency and the relaxation rate of the charge carriers. $\omega_{j}$, $\Gamma_{j}$ and $\Omega_{pj}$ are the phonon frequency, spectral width and the Lorentz oscillator strength, respectively.

In the theory calculation, we referred the parameters for the dielectric functions $\varepsilon_{\parallel}(\omega)$ and $\varepsilon_{z}(\omega)$ of CNTs in previous works measured in THz region^6,8^. Because the difference of $\varepsilon_{\parallel}(\omega)$ and $\varepsilon_{z}(\omega)$ is quite small^6^, we simplified them as the same and optimized the value to fit the experiment better. At last, the parameters used in the paper is: $\varepsilon_{c}$=3.8, $\omega_{p}$= 10 THz, $\Gamma$= 2.5 THz, $\omega_{j}$= 8 THz, $\Gamma_{j}$= 20 THz, $\Omega_{pj}$= 10 THz. And the volume fraction $f$ is set to 0.3, the thicknesses of CNT1 to CNT5 are 10.1, 13.3, 21.5, 34.4 and 69.5 μm, respectively.

**REFERENCE**

1. Nefedov, I. S., Valagiannopoulos, C. A., Hashemi, S. M. & Nefedov, E. I. Total absorption in asymmetric hyperbolic media. *Sci. Rep.* **3**, 2662 (2013).

2. Su, Z., Yin, J. & Zhao, X. Soft and broadband infrared metamaterial absorber based on gold nanorod/liquid crystal hybrid with tunable total absorption. *Sci. Rep.* **5**, 16698 (2015).

3. Pozar, D. M. *Microwave Engineering*, 4Th Ed. (John Wiley & Sons, Inc., 2011).

4. Garcı´a-Vidal, F. J., Pitarke, J. M. & Pendry, J. B. Effective medium theory of the optical properties of aligned carbon nanotubes. *Phys. Rev. Lett.* **78**, 4289-4292 (1997).

5. Bao, H., Ruan, X. & Fisher, T. S. Optical properties of ordered vertical arrays of multi-walled carbon nanotubes from FDTD simulations. *Opt. Express* **18**, 6347-6359 (2010).

6. Jeon, T.-I. Optical and electrical properties of preferentially anisotropic single-walled carbon-nanotube films in terahertz region. *J. Appl. Phys.* **95**, 5736 (2004).

7. Ugawa, A., Rinzler, A. G. & Tanner, D. B. Far-infrared gaps in single-wall carbon nanotubes. *Phys. Rev. B* **60**, 11305 (1999).

8. Parrott, E. P. J. *et al.* The use of terahertz spectroscopy as a sensitive probe in discriminating the electronic properties of structurally similar multi-Walled carbon nanotubes. *Adv. Mater.* **21**, 3953-3957 (2009).
